# Tuberculosis trends during the COVID-19 pandemic in Japan: statistical considerations and limitations

**DOI:** 10.5365/wpsar.2025.16.4.1352

**Published:** 2025-12-19

**Authors:** Keita Wagatsuma

**Affiliations:** a Division of International Health (Public Health), Graduate School of Medical and Dental Sciences, Niigata University, Niigata, Japan.; b Institute for Research Administration, Niigata University, Niigata, Japan.

Dear Editor,

I read with great interest the recent study by Kawatsu and Uchimura on the potential impact of COVID-19 on tuberculosis (TB) trends in Japan. (*1*) Using national TB surveillance data and time-series regression modelling, they estimated expected TB notifications in the absence of the pandemic. Overall, TB notifications from 2020 to 2022 fell significantly below these expectations, with notable differences by age group and place of birth. In particular, the authors should be congratulated for their effective use of nationwide surveillance data and for demonstrating an apparently good agreement between observed and fitted notifications. I commend this timely analysis but would like to discuss several points regarding the statistical methods and limitations.

First, the study analyses monthly TB notifications from 2017 to 2022, with the forecasting model trained only on the three pre-pandemic years (2017–2019). This is a relatively short baseline for capturing long-term trends and established seasonality. For time-series analysis, larger samples, such as ≥ 50 observations, are often recommended. (*2*) Also, the extension of the baseline or the incorporation of earlier historical data could improve forecast stability.

Second, the model specification and evaluation are insufficiently described, hindering the assessment of validity. (*3*) In time-series model building, choices of statistical models, such as generalized additive models, and the assumed error structure, for instance, normal, quasi-Poisson and/or negative binomial, can materially affect predictive performance. In addition, Japan’s TB notifications exhibit a clear annual cycle (summer–autumn peak) and a secular decline, but it is unclear how these components were encoded, for example, with dummies and/or splines. While mean absolute percentage error was reported, multiple metrics, such as root mean squared error and correlation coefficient, would provide a more comprehensive appraisal.

Third, the authors note that observed counts in certain subgroups differed significantly from predictions. The clarification of how significance was assessed, for instance, via prediction intervals or formal tests comparing observed and expected counts, would aid interpretation. This is particularly the case when wide, overlapping forecast confidence intervals across strata make it difficult for readers to appraise uncertainty.

Fourth, additional analytical approaches could complement the modelling strategy. Interrupted time-series analysis can quantify level and slope changes attributable to the pandemic while adjusting for pre-existing trends, seasonality, lags and autocorrelation; such models are widely used to evaluate COVID-19 impacts. (*4*)

Finally, the limitations of surveillance and the potential for underascertainment during the pandemic warrant emphasis. The analysis is ecological and cannot establish causality because the TB surveillance system is not linked to COVID-19 databases. In Japan, bacteriologically confirmed TB cases declined sharply early in the pandemic, with missed cases most likely being due to service disruption and reduced care-seeking. (*5*) Future studies linking TB and COVID-19 data, and investigating care-seeking behaviour, would help disentangle these effects.

In conclusion, Kawatsu and Uchimura’s study provides valuable evidence of the impact of COVID-19 on TB trends in Japan. With regards to the consistency of the results, their graphical presentation of observed and model-predicted counts is especially reassuring. Their conclusions may be further strengthened by addressing the above points. I thank the authors for their important contribution and hope my comments help refine understanding of the complex interplay between the COVID-19 pandemic and TB control.
